# Expression of Interleukin-17A in Lung Tissues of Irradiated Mice and the Influence of Dexamethasone

**DOI:** 10.1155/2014/251067

**Published:** 2014-03-12

**Authors:** Li-Ping Wang, Yan-Wen Wang, Bao-Zhong Wang, Gui-Ming Sun, Xiu-Yu Wang, Jun-long Xu

**Affiliations:** ^1^Department of Cadre Health, Liaocheng People's Hospital, Liaocheng 252000, China; ^2^Department of Oncology, Liaocheng People's Hospital, No. 67, Dongchang Xi Road, Liaocheng 252000, China; ^3^Department of Pathology, Liaocheng People's Hospital, Liaocheng 252000, China

## Abstract

*Purpose.* To investigate the expressions of IL-17A in different phases of radiation-induced lung injury and the effect of dexamethasone. *Methods.* The thorax of C57BL/6 mice was irradiated with 15 Gy rays. Mice from dexamethasone-treated group were injected intraperitoneally with dexamethasone (0.42 mg/kg/day) every day for the first month after irradiation. IL-17A in lung tissues was detected by immunohistochemistry. IL-17A, TGF-*β*1, and IL-6 in bronchoalveolar lavage fluid were detected by ELISA. Lung inflammation and collagen deposition were observed by H&E and Masson methods. The degree of alveolitis and fibrosis was judged according to scoring. *Results.* IL-17A expression was appreciable at 1 week, peaked at 4 weeks, and subsequently declined at 8 weeks after irradiation. IL-17A was reduced after dexamethasone application at all the observation periods. Dexamethasone also inhibited expressions of TGF-*β*, IL-6, and TNF-*α* in bronchoalveolar lavage fluid. Moreover, dexamethasone attenuated the severity of lung injury by reducing the infiltration of inflammatory cells and collagen deposition. Terms of survival and the time of death in mice of treatment group were postponed and survival rate was improved. *Conclusions.* IL-17A plays an important role in the process of radiation-induced lung injury. And dexamethasone may provide a protective role in lung injury induced by radiation.

## 1. Introduction

Radiation-induced lung injury (RILI) is one of the most common complications for patients receiving thoracic radiation. When it occurred, the dose of radiation has to be reduced. So it is one of the most important factors of reducing local control rate. Even with the most advanced three-dimensional conformal radiotherapy, there are still a considerable number of patients with varying degrees of lung injury (radiation pneumonitis and pulmonary fibrosis) [1–3]. Due to the limitation of understanding the mechanism, there have been no strategic advances in RILI treatment over the past decade.

In recent years, in-depth studies on the molecular mechanisms of RILI suggest that multicytokine-mediated cell-cell interactions play an important role in radiation pneumonitis and pulmonary fibrosis [4]. Among them, IL-17A is a newly discovered powerful proinflammatory cytokine implicated in numerous inflammatory reactions. Moreover, it can stimulate a variety of cells to release inflammatory factors and chemokines [5]. Recent studies have reported that TGF-*β*, IL6, IL-1*β*, and TNF-*α* are not only closely involved in radiation pneumonitis but also regulate IL-17A release from Th17 cells [6–8]. Th17 development and amplification and secretion of IL-17 are mainly regulated by IL-6, TGF-*β*, and TNF-*α*. Whereas TGF-*β*and IL-6 can induce Th17 activation and proliferation and IL-17 production, IL1*β* and TNF-*α* enhanced Th17 cell differentiation induced by TGF-*β* and IL-6 [8, 9]. In the model of bleomycin-induced lung injury, IL-17A gene knockout mice showed a milder lung fibrosis than gene wild-type mice [10]. Altogether, these studies suggest that IL-17A may play an important role in both pathological processes of RILI: radiation pneumonitis and pulmonary fibrosis.

A clinical study showed that dexamethasone reduces IL-17A expression in the lungs of asthmatic patients and the severity of lung inflammation [11]. However, the therapeutic potential of dexamethasone for RILI remains controversial. Animal studies showed that short-term steroid administration delays radiation pneumonitis in rats [12] and reduces radiation-related inflammation in a mouse model of RILI [13]. But in both studies, the protective effects disappeared rapidly after steroid administration was terminated. The solution may reside in the optimization of dexamethasone dosage and treatment duration. Therefore, the aim of the present study was to test the impact of low-dose long-term dexamethasone in animal models of RILI on the development of radiation pneumonitis and pulmonary fibrosis and to determine whether disease severity and treatment response correlate with changes in IL-17A expression level. This study provides further theoretical basis for glucocorticoid therapy radiation-induced lung injury and a new marker of RILI severity.

## 2. Materials and Methods

### 2.1. Animals and Reagents

Inbred male C57BL/6J mice (21 ± 2 g, 6–8 weeks) were obtained from Vital River Laboratory Animal Technology. The ELISA assay kits for mouse IL-17A, TGF-*β*1, TNF-*α*, and IL-6 were purchased from eBioscience (San Diego, CA, USA). The mouse IL-17A polyclonal Ab for immunohistochemical analysis was obtained from Abcam (Abcam Inc., Cambridge, MA, USA). Dexamethasone was purchased from Sigma (St. Louis, MO, USA).

### 2.2. Animal Groups and Intervention

The study protocols were approved by the institutional ethics committees of Liaocheng People's Hospital. Mice were kept in a group of four per cage in pathogen-free rooms and were supplied with standard laboratory diet and water. A total of 104 mice were divided into three groups randomly: (1) Sham group (*n* = 32, Sham group) which was Sham-irradiated; (2) Radiation group (*n* = 36, RT group), which was irradiated singly; (3) Dexamethasone treatment group (*n* = 36, RT + DXM group), which was treated with dexamethasone after irradiation. The treatment group was treated intraperitoneally with dexamethasone (0.42 mg/kg/day) every day for the first month after radiotherapy. Another 50 animals were used for survival study; they were divided into Sham group (*n* = 10), RT (*n* = 20), and RT + DXM group (*n* = 20). These mice were observed for 180 days following the irradiation and no animals in this cohort were euthanized.

### 2.3. Radiation Schedule

Unanesthetized mice were fixed in a plastic jig designed by ourselves which contained 12 mice at the same time. The whole thorax was irradiated by ELEKTA precise linear accelerator at a single dose of 15 Gy only once. Radiation was performed according to the characteristics as follows: beam energy: 6 MV-photons; dose rate: 3.0 Gy/min [14]; source surface distance: 1 m; size of the radiation field: 40 cm × 1.8 cm. Sham group mice were pretending to be irradiated.

### 2.4. Specimen Preparation

Mice were sacrificed at a predetermined time of 1, 4, 8, and 16 weeks after irradiation (*n* = 8 per group). The left lung was reserved for hydroxyproline analysis and the right lung for histopathology and immunohistochemical analysis. Bronchoalveolar Lavage Fluid (BALF) was collected and harvested as previously described [15].

### 2.5. Histopathological Examination

The lung tissues were fixed with 10% formalin solution, paraffin-embedded, and sectioned at an average thickness of 3 mm with a microtome. The slices obtained were stained with haematoxylin and eosin (H&E) to evaluate the inflammation and with Masson staining to identify the fibrosis in the lung. Extent of the alveolitis and pulmonary fibrosis was graded on a scale of 0 (normal) to 3 (severe) according to Szapiel's method [16].

### 2.6. Immunohistochemical Analysis

Sections obtained from paraffin-embedded tissues were placed in an antigen retrieval solution (DAKO, Denmark), followed by peroxide and protein blocking. Sections were incubated with primary antibodies specific to IL-17A (1 : 200) and then stained using a sensitive avidin-streptavidin-DAB peroxidase kit (BioGenex) according to the manufacturer's instructions. Five fields (200x) per lung specimen were examined randomly. Pictures obtained by microscope camera system were analyzed by Image-Pro Plus 6.0 image analysis system (Media Cybernetics Corporation), and integrated optical densities (IOD values) were obtained. The average IOD value of all the photos of each group represents the IOD value of the group.

### 2.7. ELISA for Cytokines in BALF

The contents of IL-17A, TGF-*β*, IL-6, and TNF-*α* in BALF were analyzed by ELISA using kits from eBioscience according to the manufacturer's instructions strictly.

### 2.8. Measurement of Lung Hydroxyproline

As a biochemical index of parenchymal collagen content, hydroxyproline was determined by alkaline hydrolysis assay. Briefly, hydroxyproline was released from lung tissue homogenates by acid hydrolysis. The hydroxyproline content of the hydrolyzation products was assessed calorimetrically at 550 nm. The results were represented as micrograms per gram lung.

### 2.9. Statistical Analysis

All statistical analyses were carried out using SPSS 16.0 statistical software. Data are represented as mean ± SD. Measurement data were analyzed by one-factor ANOVA and *q*-test between groups. Enumeration data were analyzed by chi-square test. Survival analysis used Kaplan-Meier method and the log-rank test. Results with *P* < 0.05 were considered statistically significant.

## 3. Results

### 3.1. Effect of Dexamethasone on IL-17A Expression at Various Stages of RILI

**Figure 1:**
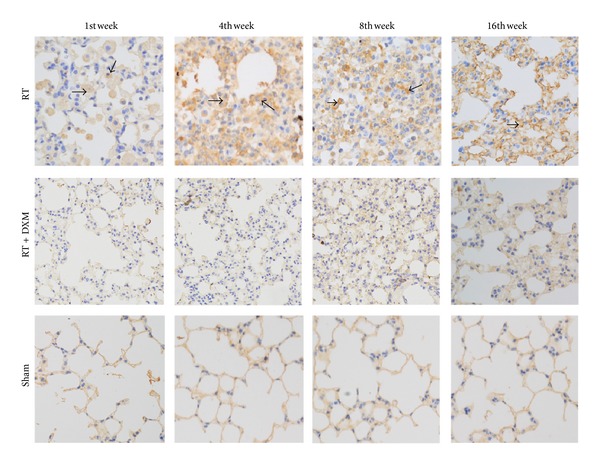
IL-17A expression in lung tissue detected by immunohistochemical method. It can be seen that cytoplasmic staining of RT group gradually deepened from the 1st week, peaked in the 4th week, declined in the 8th week, and reached a lower level in the 16th week but slightly higher than Sham group (Table 1, Figure 1). DXM application reduced mice IL-17A expression of RT group at indicated time points. The position of black arrows points to IL-17A-positive cells.

The expression of IL-17A located in the cytoplasm by immunohistochemistry. We observed a stronger expression of IL-17A by alveolar macrophages, lymphocyte, type II alveolar cells, and bronchiolar epithelium cells after radiotherapy. There are only slight expressions in lung epithelial cells for control group. The intensity of IL-17A OD values increased from 1 week to 4 weeks, then began to weaken, and sustained a higher level at 16 weeks than basal line (Figure 1). The application of dexamethasone reduced IL-17A expression at each time point of detection (Table 1, Figure 1).

**Figure 2:**
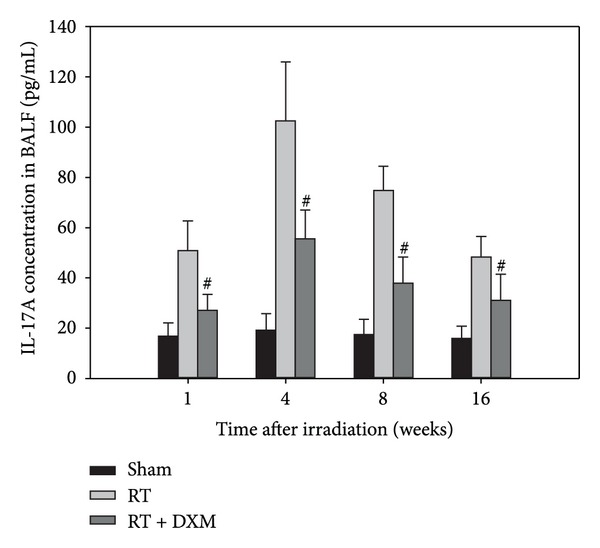
Expression of IL-17A in BALF at the indicated time points. The mice were sacrificed at the time of 1, 4, 8, and 16 weeks after irradiation, and BALF was collected for analysis of IL-17A contents. The contents of IL-17A in the BALF were analyzed by ELISA kits. Radiation stimulated an increase in the levels of IL-17A, but dexamethasone attenuated the IL-17A level in BALF. ^#^ *P* < 0.05 versus RT group at corresponding time points. Data are presented as the mean ± SD (*n* = 8/group/experiment).

The contents of IL-17A in the BALF were analyzed by ELISA kits. Radiation stimulated an increase in the levels of IL-17A in RT group, started at 1 week, peaked at 4 weeks, and dropped at 8 weeks. The results are correspondent with IL-17A immunohistochemical analysis. And dexamethasone reduced the level of IL-17A in BALF of irradiated mice (Figure 2).

### 3.2. Dexamethasone Alleviated Mouse Radiation-Related Pneumonitis and Pulmonary Fibrosis

**Figure 3:**
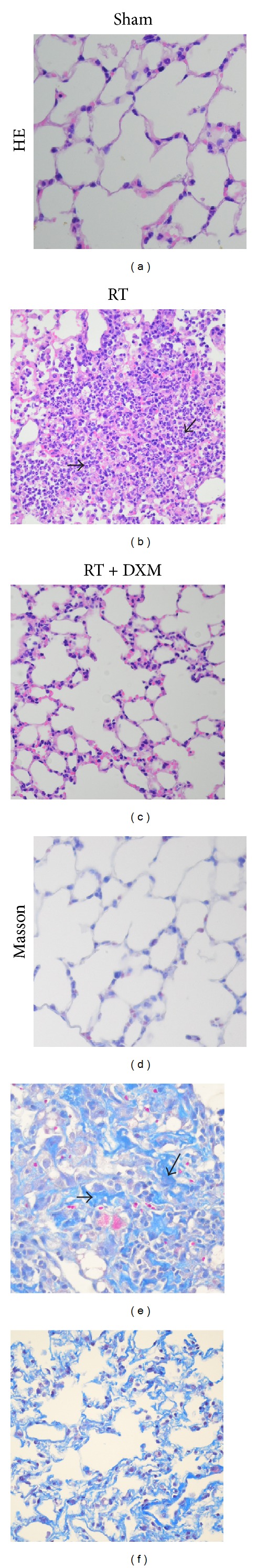
Representative samples of pathological changes in Sham group (a), (d), RT group (b), (e), and RT + DXM group (c), (f). Dexamethasone attenuates radiation-induced pneumonitis and pulmonary fibrosis. Mice were administered dexamethasone (0.42 mg/kg/day) intraperitoneally on days 1 to 30 after irradiation. Mice were sacrificed on specific time point, and lung tissue was fixed and excised into tissue sections for the detection of pulmonary inflammation and collagen deposition by H&E or Masson staining. In (a), (b), and (c), dexamethasone promoted the resolution of the radiation-induced pulmonary inflammation in the 4th week as indicated by H&E staining of the lung sections. (a) Sham group. (b) RT group, significant radiation pneumonitis and inflammatory cell infiltration (black arrows position). (c) RT + DXM group, inflammatory cell infiltration was significantly reduced without significant leakage. In (d), (e), and (f), dexamethasone promoted the resolution of the radiation-induced pulmonary fibrosis in the 16th week as indicated by Masson staining of the lung sections. (d) Sham group. (e) RT group, fibrosis stage, a large number of collagen depositions (black arrow position). (f) RT + DXM group, collagen deposition was significantly reduced compared with RT group; the alveolar structure is relatively intact.

**Figure 4:**
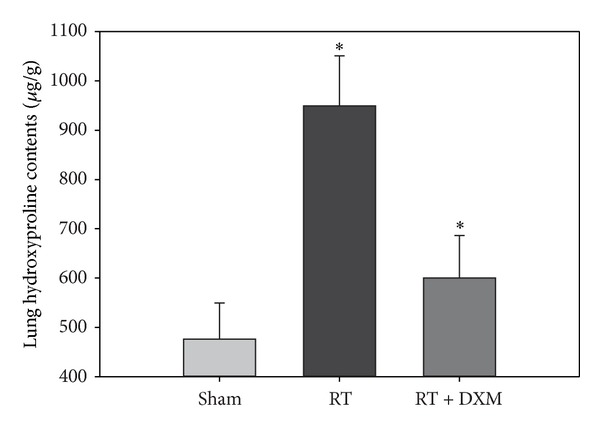
Content of lung hydroxyproline was determined by alkaline hydrolysis assay. Hydroxyproline content was significantly higher at 16 weeks than Sham group. Dexamethasone reduced lung hydroxyproline content which can reflect the content of collagen. *P < 0.001 versus RT group and Sham group at corresponding time points. Data are presented as the mean ± SD (*n* = 8/group/experiment).

Alveolitis is the main lesion before 8 weeks after irradiation, and since then the degree of alveolitis began to gradually be mild, lung parenchyma structural damage, disorder, and focal fibrosis began to appear, and it gradually evolved into fibrosis. We examined hydroxyproline content of mice lung tissue at 16 weeks and found that of RT group significantly higher than the Sham group (*P* < 0.001). Hydroxyproline content was decreased significantly after dexamethasone treatment (*P* < 0.001). This result is consistent exactly with the pathological changes (Figures 3(e) and 3(f)). The study found that dexamethasone can reduce the tissue collagen deposition (Figures 3(e) and 3(f)) and decrease the tissue content of hydroxyproline (Figure 4) compared with RT group. In addition, administration of dexamethasone reduced the degree of alveolitis and fibrosis significantly in mice according to Szapiel's scales. That is to say, the lung injury of DXM treatment group is milder than that of radiation alone group. (Figure 3 and Table 2) (*P* < 0.001).

### 3.3. Dexamethasone Reduced the Level of Cytokines Closely Related to Radiation Pneumonia and Fibrosis

The level of TNF-*α*, IL-6, and TGF-*β*1 was higher significantly after irradiation than the Sham group, which is consistent with previous literature results [17, 18]. After a study on mouse lung tissue and lavage fluid, we found that dexamethasone can reduce inflammatory cell infiltration (Figures 3(b) and 3(c)) and decrease inflammatory cytokines related to lung fibrosis closely such as TNF-*α*, IL-6, and TGF-*β*1 protein expression in BALF in the 4th week (Table 3), compared with the RT group.

### 3.4. Effect of Dexamethasone on Survival Rate of Mice Undergoing Thoracic Radiation

**Figure 5:**
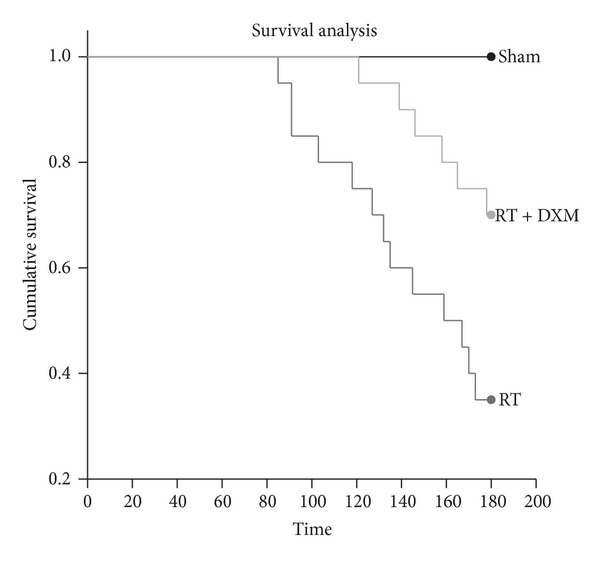
Effect of dexamethasone on the survival of mice after irradiation. C57BL/6J mice were treated with a single dose (15 Gy) to the entire thorax. Dexamethasone treatment increased the survival rates of irradiated mice. The survival rates at 180 days in Sham (*n* = 10), RT group (*n* = 20), and RT + DXM group (*n* = 20) are 100.0%, 35.0%, and 70.0%, respectively (RT versus RT + DXM group, *P* = 0.0323). The survival rate was analyzed using the Kaplan-Meier method.

Mice began to die on the 85th day after irradiation; dexamethasone applications delayed the time of death in mice and improved the survival rate of mice receiving total thoracic irradiation. In the radiation group, 13 mice (65.0%) died within 180 days following the radiation. Only 6 mice (30.0%) of dexamethasone treatment group died on days 121, 139, 146, 158, 165, and 178, respectively. The survival rate in the treatment group was higher than in the radiation group (*P* = 0.0323; Figure 5). Dexamethasone applications delayed the time of death and improved the survival rate of mice RILI.

## 4. Discussion

Previous studies proved that IL-17A was significantly increased in a variety of chronic inflammatory disease models, and blocking IL-17A signals can relieve inflammation and fibrosis, thus confirming that IL-17A plays an important role in inflammatory processes [10, 19, 20]. Wilson et al. found that the severity of lung injury in mice was significantly decreased after mice IL-17A gene knockout, which also showed the importance of IL-17A in inflammation and fibrosis [10]. However, it is not yet reported whether IL-17A plays a role in radiation-induced lung injury.

In this study, the model of RILI was established through C57BL/6 mice irradiated with 15 Gy in the chest. Immunohistochemistry and ELISA methods were used to detect IL-17A in the lung tissue and BALF. The results showed that expression of IL-17A was elevated gradually in lung tissue after irradiation, peaked in the fourth week, and began to decline in the eighth week. This trend is consistent with IL-17A content in BALF. The result implies that IL-17A may be another important cytokine involved in RILI in addition to IL-6, TGF-*β*, and TNF-*α*. Dexamethasone treatment decreased expression of IL-17A in both lung tissues and BALF; cytokines IL-6, TGF-*β*, and TNF-*α* were reduced too. So it seems that dexamethasone eased the severity of acute inflammation and subsequent fibrosis induced by these cytokines. We examined the content of hydroxyproline in lung tissues in the 16th week and found that it increased more significantly in lung tissue irradiated mice than that of the control group (*P* < 0.001). After dexamethasone treatment, mice hydroxyproline content decreased significantly (*P* < 0.001). This result is consistent exactly with the pathological changes. In terms of survival, the application of dexamethasone delayed the time of irradiated mice death and increased survival time of mice irradiated group.

Recent studies suggest that the incidence of radiation-induced pulmonary fibrosis is not simply the result of chronic outcome of radiation pneumonitis. In fact, in the early radiation injury, along with the radiation pneumonitis, fibrosis has been launched [21]. Traditional idea is that dexamethasone only takes effect in the early phases of radiation-induced lung injury. So we only use dexamethasone treatment in the early phases of the experiment (within 1 month after irradiation). Different from previous studies, we employ a low-dose and long treatment duration of dexamethasone to interfere with radiation-induced lung injury in mice. The results showed that dexamethasone to some extent alleviated the severity of radiation pneumonitis, thereby weakening pulmonary fibrosis.

Biological effects of IL-17A have not been reported in RILI, and the exact mechanism is unclear. However, IL-17A contribution to idiopathic or drug-induced pulmonary fibrosis can be summarized as TGF-*β* dependent and TGF-*β* independent roles [10, 19, 22]. Studies have shown that, in a mouse model of asthma, dexamethasone inhibited gene expression of IL-17A and alleviated the exacerbation of the inflammatory process induced by IL-17A [23]. In this study, long-term and low-dose dexamethasone inhibited the expression of IL-17A and TGF-*β*, blocked both TGF-*β* dependent and independent ways, moderated the biological effects in RILI, and thereby played a protective role in lung tissue.

Basic pharmacological studies confirmed that dexamethasone can regulate gene transcription and expression of a variety of inflammatory cytokines [24], such as IL-6, TNF-*α*, and TGF-*β*, which is closely related to radiation-induced lung fibrosis [25–28]. TGF-*β* is considered as the most potent of the profibrotic factors and also proved to be the most important cytokines in the process of radiation fibrosis. Recent studies have shown that glucocorticoids inhibit TGF-*β* activity not only through mitogen-activated protein kinases (MAPKs) but also through nuclear transcription factor (NF-*κ*B) [29, 30].

In summary, IL-17A may play an important role in the process of radiation-induced lung injury, especially in the early phase. Dexamethasone applications attenuate the severity of radiation pneumonitis and pulmonary fibrosis, by reducing inflammatory and fibrogenic cytokine expression, thereby enhancing the survival time of irradiated mice. Indeed, the further experimental studies should be carried out by using IL-17A knockout mice or IL-17A antagonist to study radiation pneumonitis and fibrosis, thus establishing the direct evidence of IL-17A in radiation-induced lung injury.

## Figures and Tables

**Table 1:** The average optical density of IL-17A analysis and comparison in each group (*n* = 5, mean ± SD).

Group	1 w	4 w	8 w	16 w
Sham	504.81 ± 65.03	473.84 ± 76.30	488.18 ± 84.83	433.70 ± 69.51
RT^#^	1056.20 ± 234.80	2664.31 ± 596.54	1890.94 ± 245.38	1322.10 ± 201.45
RT + DXM^##^	670.60 ± 176.59	1360.36 ± 352.87	1037.04 ± 148.53	889.21 ± 123.63

^##,#^ *P* < 0.05 versus Sham group; ^##^ *P* < 0.05 versus RT group at corresponding time point.

**Table 2:** Comparison of the pathology grade of alveolitis and pulmonary fibrosis.

Lung injury	Group	*n*	Grade			
			0	I	II	III
Alveolitis	Sham	24	21	3	0	0
	RT	24	0	3	10	11
	RT + DXM	24	0	17*	3*	4*

Lung fibrosis	Sham	8	8	0	0	0
	RT	10	0	1	7	2
	RT + DXM	12	0	8^#^	3^#^	0

Alveolitis: *P < 0.05 compared with RT group.

Lung fibrosis: ^#^ *P* < 0.05 compared with RT group within the same grading category.

**Table 3:** The content of TGF-*β*1, IL-6, and TNF-*α* in BALF in each group (*n* = 8, mean ± SD).

Group	TGF-*β*1 (pg/mL)	IL-6 (pg/mL)	TNF-*α* (pg/mL)
Sham	68.45 ± 18.18	22.42 ± 6.54	16.39 ± 6.95
RT	320.78 ± 69.30	88.41 ± 14.78	62.76 ± 11.49
RT + DXM	121.89 ± 26.43*	40.14 ± 13.14*	30.25 ± 10.91*

*P < 0.001 versus RT group.
